# Effects of starvation-induced negative energy balance on endoplasmic reticulum stress in the liver of cows

**DOI:** 10.5713/ab.21.0140

**Published:** 2021-06-23

**Authors:** Md Aminul Islam, Shuya Adachi, Yuichiroh Shiiba, Ken-ichi Takeda, Satoshi Haga, Shinichi Yonekura

**Affiliations:** 1 Department of Biomedical Engineering, Graduate School of Medicine, Science and Technology, Shinshu University, Kamiina, Nagano 399-4598, Japan; 2 Department of Biomedical Engineering, Graduate School of Science and Technology, Shinshu University, Kamiina, Nagano 399-4598, Japan; 3 Faculty of Agriculture, Shinshu University, Kamiina, Nagano 399-4598, Japan; 4 Grazing Animal Unit, Division of Grassland Farming, Institute of Livestock and Grassland Science, NARO, Nasushiobara, Tochigi 329-2793, Japan; 5 Department of Biomolecular Innovation, Institute for Biomedical Sciences, Shinshu University, Kamiina, Nagano 399-4598, Japan

**Keywords:** Dairy Cow, Endoplasmic Reticulum Stress, Fasting, Liver, Negative Energy Balance

## Abstract

**Objective:**

Endoplasmic reticulum (ER) stress engages the unfolded protein response (UPR) that serves as an important mechanism for modulating hepatic fatty acid oxidation and lipogenesis. Chronic fasting in mice induced the UPR activation to regulate lipid metabolism. However, there is no direct evidence of whether negative energy balance (NEB) induces ER stress in the liver of cows. This study aimed to elucidate the relationship between the NEB attributed to feed deprivation and ER stress in bovine hepatocytes.

**Methods:**

Blood samples and liver biopsy tissues were collected from 6 non-lactating cows before and after their starvation for 48 h. The blood non-esterified fatty acids (NEFA), β-hydroxybutyric acid (BHBA) and glucose level were analyzed. Real-time quantitative polymerase chain reaction and Western blotting were used to explore the regulation of genes associated with UPR and lipid metabolism.

**Results:**

The starvation increased the plasma BHBA and NEFA levels and decreased the glucose level. Additionally, the starvation caused significant increases in the mRNA expression level of spliced X-box binding protein 1 (*XBP1s*) and the protein level of phosphorylated inositol-requiring kinase 1 alpha (p-IRE1α; an upstream protein of XBP1) in the liver. The mRNA expression levels of peroxisome proliferator-activated receptor alpha and its target fatty acid oxidation- and ketogenesis-related genes were significantly upregulated by the starvation-mediated NEB. Furthermore, we found that the mRNA expression levels of lipogenic genes were not significantly changed after starvation.

**Conclusion:**

These findings suggest that in the initial stage of NEB in dairy cows, the liver coordinates an adaptive response by activating the IRE1 arm of the UPR to enhance ketogenesis, thereby avoiding a fatty liver status.

## INTRODUCTION

The liver is the central tissue maintaining metabolic homeostasis as the animal shifts between fed and fasted states. During fasting, the animal mobilizes its body fat reserves to provide non-esterified fatty acids (NEFAs) as an energy source to fulfill its energy requirements. In the liver, NEFAs can be oxidized by mitochondria or re-esterified to form triglycerides (TG) within the endoplasmic reticulum (ER) to be stored in lipid droplets or secreted/exported into the blood as a component of very-low-density lipoprotein [1,2]. Fatty acid oxidation is an important mechanism for maintaining hepatic lipid homeostasis under fasted state. Impaired fatty acid oxidation leads to abnormal accumulation of TG in the liver and results in hepatic steatosis [3,4]. Moreover, when the excessive uptake of NEFAs by the liver cannot be accommodated through fatty acid oxidation and lipid secretion, ketosis or a fatty liver ensues in dairy cows [5]. Importantly, the molecular mechanisms behind the detrimental effects of negative energy balance (NEB) as well as NEFAs on the liver of dairy cows have not been fully elucidated.

The ER is a subcellular organelle that is important for ensuring the smooth function of synthetic pathways, including lipogenesis. Any perturbation of its homeostasis causes ER stress, resulting in the accumulation of unfolded proteins on the organelle. Such scenario activates the unfolded protein response (UPR), which can occur through three arms: PKR-like endoplasmic reticulum kinase (PERK), inositol-requiring kinase 1 (IRE1), and activating transcription factor 6 (ATF6). UPR activation alleviates ER stress, however, unresolved or prolonged ER stress induces apoptosis [6]. Recent accumulated data suggested that ER stress in liver leads to substantial and coordinated regulation of gene involved in lipid metabolism [7,8]. The UPR serves as an important mechanism for modulating hepatic fatty acid oxidation and lipogenesis [9,10]. Indeed, ER stress is considered as key factor in hepatic steatosis [11]. Furthermore, chronic fasting conditions in mice have shown that it has been demonstrated that chronic fasting in mice induced the UPR activation to regulate lipid metabolism [12,13]. Therefore, we hypothesized that an increased amount of serum NEFAs attributed to NEB would exert ER stress in bovine liver cells. However, none of these studies had examined the direct relationship between NEB and ER stress in dairy cows.

Therefore, our objective was to elucidate the relationship between the NEB attributed to feed deprivation and ER stress in bovine hepatocytes. To this end, we observed whether an NEB state affects the expression of ER stress-related genes and proteins in the liver of starved dairy cows. We also measured the expression of genes related to oxidation, ketogenesis and lipogenesis following starvation. Therefore, this NEB and ER study will play a key role in our better understanding of the liver physiology of dairy cows in the state of NEB.

## MATERIALS AND METHODS

### Ethics statement

All animal related procedures in this study were approved by the Committee for Animal Experiments of Shinshu University (Approval No. 020019).

### Animals and sampling

Six nonpregnant, nonlactating Holstein cows were enrolled in the study. The average age of cows was 4.7 yr, and average body weight (BW) and body condition score [14] were 735 kg and 3.5, respectively. Cows were maintained in individual tie stalls.

Before 48 h fasting, cows were fed a constant amount of grass hay (about 1.0% of BW/d, DM basis) twice daily (0930 and 1630). Animals had free access to water and a trace mineralized salt block throughout the experimental period.

Blood samples were collected from the tail veins at d 0 (before fasting, 0900 h) and d 2 (after 48 h fasting). After sampling, blood samples (heparin tube) were immediately cooled on crushed ice. After centrifugation (1,000×*g* for 20 min at 4°C), separated plasma was immediately frozen and stored at −30°C until analyses. Plasma concentrations of NEFA, β-hydroxybutyric acid (BHBA), and glucose were determined using a clinical biochemistry autoanalyzer (BioMajesty JCA-BM 6050; JEOL Ltd., Tokyo, Japan).

Liver biopsies were performed at d 0 (before fasting) and d 2 (after 48 h fasting), following a procedure described previously [15]. Liver samples were rinsed with ice-cold saline solution and immediately frozen in liquid nitrogen. Samples were stored at −80°C until gene and protein expression analyses.

### Quantitative real-time polymerase chain reaction

The entire RNA was extricated from liver samples applying TRIzol (Invitrogen, Carlsbad, CA, USA) maintaining the protocol given by the manufacturing company. cDNA was synthesized from gross RNA utilizing gDNA Remover requiring qPCR RT Master Mix (Toyobo, Osaka, Japan). SYBR Premix Ex Taq II (TaKaRa Biotechnology, Kusatsu, Japan) was applied for quantitative real-time polymerase chain reaction (PCR) analysis. Primer sequences are shown in Table 1. 2^−ΔΔCT^ comparative method was used for quantification of relative gene expressions and was expressed as values relative to control (d 0). The used house-keeping gene were β-actin and ribosomal protein S9. The amplification of serial cDNA dilutions was performed to examine the sensitivity of reactions and magnification of contaminating products, such as extensions of self-annealed primers. Manufacturer’s instructions were maintained to perform data analysis.

### Western blot analysis

Total proteins were extracted from liver samples and prepared for Western blotting according to the manufacturer’s instructions (Minute Total Protein Extraction Kit; Invent Biotechnology, Inc., Plymouth, MN, USA). Protein concentrations were evaluated using a Bio-Rad Protein Assay Kit (Bio-Rad Laboratories, Hercules, CA, USA). The samples were size-fractionated using sodium dodecyl sulfate-polyacrylamide gel electrophoresis and polyvinylidene difluoride (PVDF) membranes were used to transfer the protein from gel to membrane. Afterwards, membranes were incubated with anti-phosphorylated PERK (Santa Cruz Biotechnology, Santa Cruz, CA, USA) and anti-α-tubulin (MBL Co., Nagoya, Japan) antibodies diluted in blocking buffer. Next, anti-rabbit IgG secondary antibody (GE Healthcare, Pittsburgh, PA, USA) was used for incubation of the membranes. An ECL Prime Western Blotting Detection Reagent Kit (GE Healthcare, USA) was utilized for visualization of the enhanced chemiluminescent (ECL) membranes and images were captured using an Image Quant LAS 500 (GE Healthcare, USA). The images were analyzed with the ImageJ software from the NIH.

### Statistical analysis

For all the experiments in this study, paired Student’s *t*-test was utilized to measure the statistical significance to make a comparison between two samples. Data were expressed as mean±standard error of the mean. Statistically significant was considered at p<0.05.

## RESULTS

### Effects of 48-h starvation on plasma indicators of negative energy balance in non-lactating dairy cows

**Figure 1:**
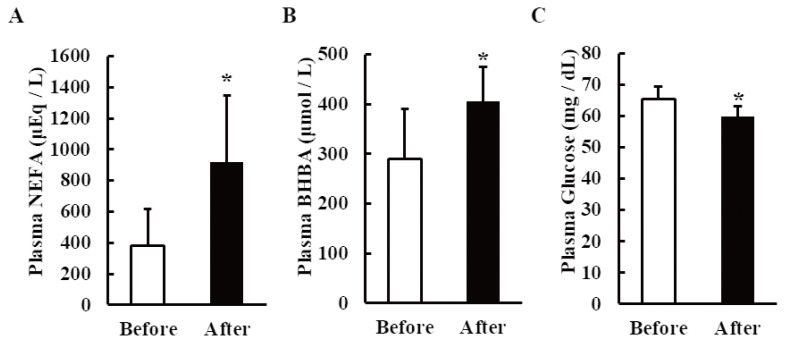
Effects of 48-h starvation on plasma indicators of negative energy balance in non-lactating dairy cows. Plasma concentrations of (A) non-esterified fatty acids (NEFA), (B) β-hydroxybutyric acid (BHBA), and (C) glucose before and after starvation. The results are expressed as the mean values±standard error of the mean (n = 6). * p<0.05 compared with before starvation.

The starvation significantly increased the BHBA and NEFA levels (p<0.05) and decreased the glucose level significant (p<0.05) (Figure 1).

### Hepatic mRNA expression levels in non-lactating cows before and after their starvation

**Figure 2:**
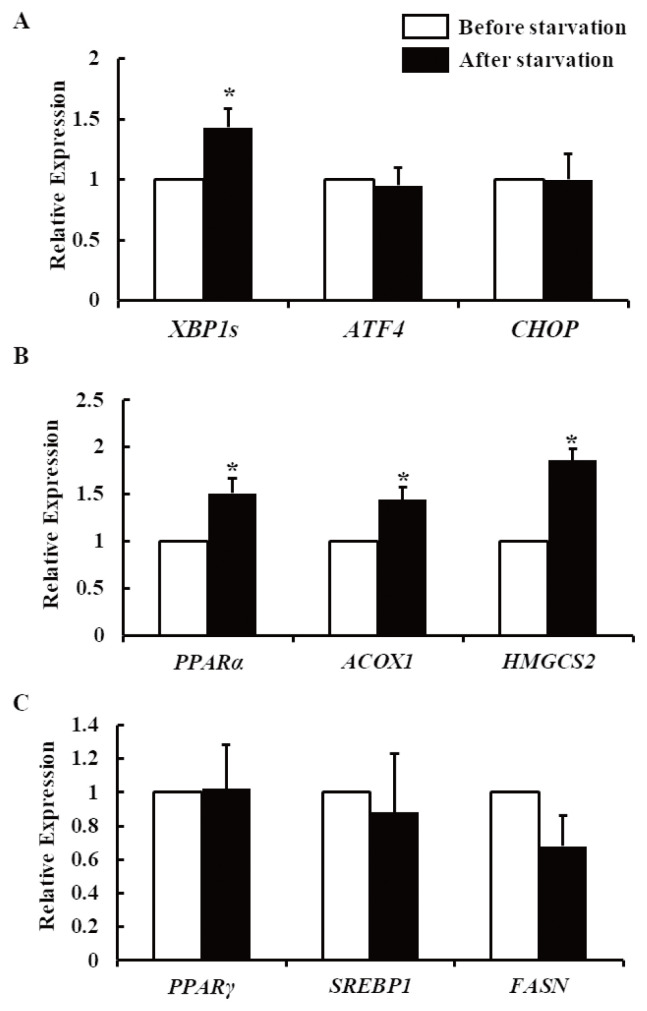
Hepatic mRNA expression levels in non-lactating cows before and after their starvation for 48 h. (A) mRNA expression of *XBP1s*, *ATF4*, and *CHOP*. (B) mRNA expression of *PPARα*, *ACOX1*, and *HMGCS2*. (C) mRNA expression of *PPARγ*, *SREBP1*, and *FASN*. The results are expressed as the mean values±standard error of the mean (n = 6). *XBP1s*, spliced X-box binding protein 1; *ATF4*, activating transcription factor 4; *CHOP*, C/EBP homologous protein; *PPAR*, peroxisome proliferator-activated receptor; *ACOX1*, acyl-CoA oxidase 1; *HMGCS2*, 3-hydroxy-3-methylglutaryl-CoA synthase 2; *SREBP1*, sterol regulatory element-binding protein 1; *FASN*, fatty acid synthase. * p<0.05 compared with before starvation.

Next, the mRNA expression levels of UPR-related genes in the liver tissue before and after 48-h starvation were analyzed by real-time PCR. Whereas the expression of X-box binding protein 1 (*XBP1s*) was increased after the starvation period, there was no difference found in the activating transcription factor 4 (*ATF4*) and C/EBP homologous protein (*CHOP*) levels (Figure 2A). Moreover, the expression levels of peroxisome proliferator-activated receptor α (PPARα) and its target genes for fatty acid oxidation and ketogenesis (i.e., acyl-CoA oxidase 1 [*ACOX1*] and 3-hydroxy-3-methylglutaryl-CoA synthase 2 [*HMGCS2*]) were increased after starvation (Figure 2B). In the case of *PPARγ* (a regulator of lipogenesis) and the lipogenic genes sterol regulatory element-binding protein 1 (*SREBP1*) and fatty acid synthase (*FASN*), their expression levels were not notably different before and after starvation (Figure 2C).

### The starvation significantly increased the phosopho-IRE1**α** expression

**Figure 3:**
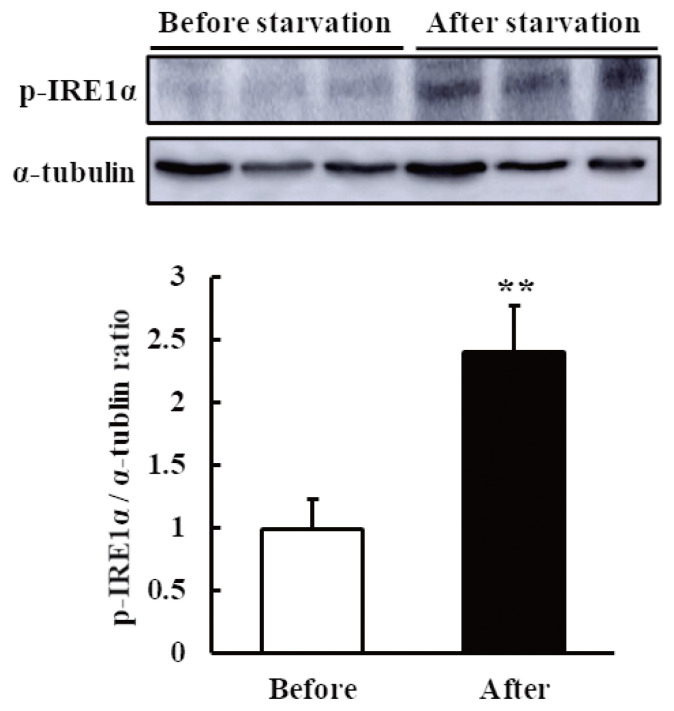
Protein levels of phosphorylated inositol-requiring kinase 1 alpha (p-IRE1α) in the liver of non-lactating cows before and after their starvation for 48 h. The p-IRE1α and α-tubulin (internal control) levels were detected by western blot assay. The quantification of the p-IRE1α levels is shown in the bar graph. The results are expressed as the mean values±standard error of the mean (n = 6). ** p<0.01 compared with before starvation.

To confirm whether the IRE1–XBP1 arm of the UPR is activated after starvation, we analyzed the protein expression of p-IRE1α (an upstream protein of XBP1) by western blot assay. The results showed that p-IRE1α was increased in the liver tissue after the starvation period (Figure 3).

## DISCUSSION

In this study, the significant increase in NEFAs and BHBA and decrease in the glucose level in the blood of the dairy cows after 48 h of starvation (Figure 1) reflected the mobilization of adipose tissue reserves for maintaining the energy requirements of the animal. The blood NEFA and BHBA levels depend on the degree of NEB. Mohamed et al. used the starvation model to study the metabolic profile of blood in fasted dairy cows and showed that the NEFA level was more than two times higher after 4 days of starvation [16]. By contrast, the BHBA levels in portal, hepatic, and jugular blood rose to 197%, 190%, and 186% of the pre-fasting levels, respectively. The mild increase in BHBA in that study suggested the ability of non-lactating cows to resist the development of hyperketonemia during food deprivation. Hence, the increase in serum NEFA and BHBA levels after 48 h of starvation confirmed that the dairy cows had entered into an NEB state in our study.

The ER stress induction of the UPR and the downstream signaling molecules by specific arms have been examined in cell culture and animal models to elaborate their functions and roles in lipid metabolism. The PERK–eukaryotic initiation factor 2 alpha (eIF2α)–ATF4 pathway was shown to enhance lipogenesis and hepatic steatosis. ATF4 knockout was found to protect against diet-induced obesity, hypertriglyceridemia, and hepatic steatosis by significantly decreasing the expression of lipogenic genes, such as *PPARγ*, *SREBP-1c*, and *FASN* [17]. Interestingly, we found that the starvation-induced NEB did not activate the PERK arm of the UPR, as the *ATF4* and *CHOP* mRNA expression levels were not affected by starvation (Figure 2A). Consistent with the levels of *ATF4* expression, those of *PPARγ* (a master regulator of lipogenesis) and the lipogenic genes *SREBP1* and *FASN* were also not upregulated after starvation (Figure 2C). Our findings are supported by another study in which fasted mice showed no upregulation of *PPARγ* expression in the liver [12]. However, contrary to our assumption that an extended starvation period may lead to lipogenesis in the liver, the NEB mediated by 48 h of starvation did not cause this process in the non-lactating dairy cows.

**Figure 4:**
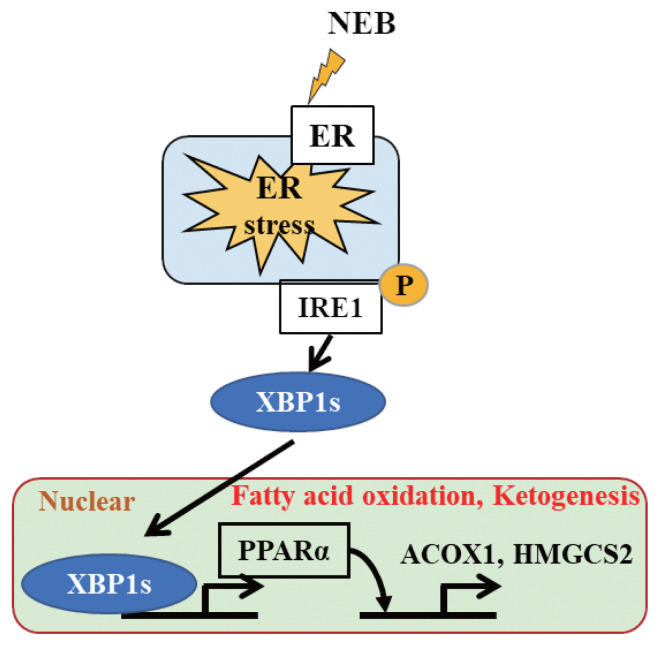
Schematic diagram depicting how a starvation-induced negative energy balance (NEB) state promotes the adaptive response in the liver, that is, by activating the IRE1–XBP1 pathway, which triggers PPARα and its target genes to enhance fatty acid oxidation and ketogenesis. *PPARα*, peroxisome proliferator-activated receptor α.

The hepatic expression of *XBP1s* was significantly higher after starvation (Figure 2A), leading to an increase in p-IRE1α protein expression (Figure 3). It has been reported that the IRE1–XBP1 pathway is required for the maintenance of hepatic lipid homeostasis under ER stress conditions, acting as a nutrient sensor during starvation and stimulating ketogenesis through the XBP1s–PPARα axis in mice [12]. In that study, the specific knockout of IRE1α in the liver reduced the expression of PPARα, ACOX1, and HMGCS2. Consistent with the finding in that mouse study, starvation activated hepatic *PPARα* and its target genes *ACOX1* and *HMGCS2* in our study (Figure 2B). The first step of peroxisomal beta-oxidation is carried out by ACOX1. Robust ketogenesis is limited to the hepatocytes owing to the relatively restricted expression of the fate-committing ketogenic enzyme HMGCS2 under normal conditions [18]. A failure in ketone body metabolism could potentially contribute to fatty liver disease. According to Cotter et al [19], chow-fed mice that were rendered devoid of HMGCS2 produced adequate hepatic metabolites suitable for lipogenesis because of their inability to undergo ketogenesis. The same mice suffered from hepatic injury as a result of excessive lipogenesis during their intake of a high-fat diet, indicating that ketogenesis is used by the animals to prevent the lipogenic process. Therefore, the NEB induced by starvation promoted an adaptive response, inducing fatty acid oxidation and ketogenesis instead of lipogenesis in the liver by activating the IRE1–XBP1 pathway (Figure 4). At the same time, the activation of only the IRE1 arm of the UPR indicated that the level of ER stress was mild and tolerable by the hepatocytes, allowing normal physiological development to occur. Otherwise, severe ER stress would have led to the pathogenesis of liver diseases by altering cellular developmental signals and gene expression [20]. Overall, the 48-h starvation resulted in only mild ER stress, activating the IRE1 arm of the UPR only, thereby allowing the liver to adapt via lipid oxidation and ketogenesis.

In the transition period, bovine hepatocytes experience severe ER stress together with an upregulation in lipogenic gene expression, resulting in periparturient diseases (e.g., severe fatty liver) and an observed increase in the protein expression levels of phosphorylated -PERK, p-IRE1, and ATF6 [21,22]. Previous studies suggested that severe ER stress was occurred in bovine mammary glands during transition period [23] and sever ER stress and lipogenesis were observed to occur simultaneously in the mammary epithelial cells of cows with ketosis [24]. Furthermore, accumulating evidence suggests that excessive fatty acids cause severe ER stress to hepatocytes and bovine mammary epithelial cells *in vitro* [22,25]. It is speculated that a similar adaptive response to NEB may occur during the periparturient period, However, NEB caused by starvation is not comparable to NEB induced in early lactation as endocrine-immune-metabolic responses are markedly different. Therefore, further study focusing on early lactation will be needed to understand the relationship between NEB induced in early lactation and hepatic ER stress.

Instead of inducing lipogenesis or severe ER stress, the 48-h starvation induced the initial adaptive response to NEB by enhancing ketogenesis and preventing serious ER stress. Our findings provide an insight into the initial molecular mechanisms that the liver of periparturient dairy cows adopt to avoid hepatic diseases ensuing from the NEB state.

## Figures and Tables

**Table 1:** Sequences of primers used for real-time polymerase chain reaction amplification

Gene	Primers (5′ to 3′)
*XBP1s*	Forward TGCTGAGTCCGCAGCAGGTG Reverse GCTGGCAGACTCTGGGGAAG
*ATF4*	Forward CCGAGATGAGCTTTCTGAGC Reverse AGCATCCTCCTTGCTGTTGT
*CHOP*	Forward CTGAAAGCAGAGCCTGATCC Reverse GTCCTCATACCAGGCTTCCA
*PPARα*	Forward CGGTGTCCACGCATGTGA Reverse TCAGCCGAATCGTTCTCCTAAA
*ACOX1*	Forward CCATTGCCGTCCGATACAGT Reverse GTTTATATTGCTGGGTTTGATAATCCA
*HMGCS2*	Forward GCCCAATATGTGGACCAAAC Reverse ATGGTCTCAGTGCCCACTTC
*PPARγ*	Forward CCAAATATCGGTGGGAGTCG Reverse ACAGCGAAGGGCTCACTCTC
*SREBP1*	Forward ATGCCATCGAGAAACGCTAC Reverse GTCCGCAGACTCAGGTTCTC
*FASN*	Forward GCATCGCTGGCTACTCCTAC Reverse GTGTAGGCCATCACGAAGGT
*ACTB*	Forward CATCGCGGACAGGATGCAGAAA Reverse CCTGCTTGCTGATCCACATCTGCT
*RPS9*	Forward CCTCGACCAAGAGCTGAAG Reverse CCTCCAGACCTCACGTTTGTTC

*XBP1s*, spliced X-box binding protein 1; *ATF4*, activating transcription factor 4; *CHOP*, C/EBP homologous protein; *PPARα*, peroxisome proliferator-activated receptor α; *ACOX1*, acyl-CoA oxidase 1; *HMGCS2*, 3-hydroxy-3-methylglutaryl-CoA synthase 2; *SREBP1*, sterol regulatory element-binding protein 1; *FASN*, fatty acid synthase; *ACTB*, β-actin; *RPS9*, ribosomal protein S9.
